# Acceptance of Caregiver–Patient Support to Latinx Coping with Advanced Cancer (CASA) Intervention: A Caregiver Case Study

**DOI:** 10.3390/ijerph20064996

**Published:** 2023-03-12

**Authors:** Lianel Rosario-Ramos, Cristina Peña-Vargas, Normarie Torres-Blasco

**Affiliations:** 1 School of Behavioral and Brain Sciences, Ponce Health Sciences University, Ponce 00716, Puerto Rico; 2 Ponce’s Research Institute, Ponce Health Sciences University, Ponce 00716, Puerto Rico

**Keywords:** Latino, Latinx, advanced cancer, informal caregiver, meaning, meaning centered psychotherapy

## Abstract

Latinos frequently assume caregiver roles when the need arises in their social nucleus. Because of their active role, caregivers are heavily involved in their family member’s cancer trajectory. Therefore, there is a need for culturally adapted interventions that integrate the caregiver and cancer patient. The objective is to present a case study of a former caregiver’s experience and acceptance of the cultural adaptation of Caregiver–Patient Support to Latinx Coping with Advanced Cancer (CASA) intervention. We conducted a case study with a male caregiver between the ages of 20 and 30. A male caregiver expressed his experience and acceptance of a psychosocial intervention. He conveyed moderate to high acceptance of intervention components through anecdotes and opinions based on his experiences as a caregiver for multiple family members. Finally, he reported distress, but he presented little to no symptoms of caregiver burden, depression, anxiety, and hopelessness. It is crucial to culturally adapt interventions that integrate caregivers when they play a big part in their loved one’s cancer journey. Integrating their perspective when adapting an intervention may assist in providing essential information that will benefit the patient and caregiver.

## 1. Introduction

Informal caregivers are family members who take care of loved ones once diagnosed with an illness [1]. Latino caregivers are mostly family members because many believe family members should be responsible for providing care [2]. Informal caregivers typically set aside their physical and emotional needs when assuming a caregiving role to attend to their loved ones [3]. As a result, a cancer diagnosis not only affects the patient but also has an impact on the caregiver [4,5]. Caregiving is a demanding job that requires the family member to provide physical and emotional support [6] and financial support [7] and plays an active role in cancer treatment and end-of-life decision-making [8,9]. However, there is a significant difference between Latino caregivers and other ethnic groups.

On average, Latino caregivers are younger, are married, and care for children under 18 while caring for and living with their loved ones [10,11]. Additionally, they dedicate more hours per week, face higher-intensity caregiving situations, and report more physical and financial strain [10,11]. Overall, Latino caregivers have experienced a decline in health status [10]. Due to the stressful demand of caregiving, caregivers may experience physical problems, psychological distress, and changes to the overall quality of life (QOL) [12,13]. As a result, caregivers participate in dyadic or caregiver-aimed interventions [14,15,16].

Meaning-centered therapy (MCP) is a psychotherapeutic intervention that focuses on the meaning of life, the will to find meaning, freedom of will, and sources of meaning. MCP is based on Viktor Frankl’s existential model, which underscores how meaning is a primary source of human motivation and can be found during adverse situations. This intervention is aimed at advanced cancer patients so they can find or enhance their sense of meaning while facing advanced cancer [17,18]. The literature shows the effectiveness of culturally adapted mental health services and interventions [19,20,21,22]. To mitigate an existing gap regarding Latinos, Rosario Costas-Muñiz is adapting MCP for Latino advanced cancer patients (MCP-L) [23,24,25]. Some findings indicated a need to integrate family and communication skills to adapt to the cancer diagnosis [26]. As a result, Normarie Torres-Blasco is developing an adaptation that integrates the caregiver and communication skills for both patient and caregiver as a dyadic. Simultaneously with the MCP-L’s adaptation process, an adaptation with caregivers was developed and tested. MCP-C is an adaptation focusing on the caregiver’s existential distress and meaning [27,28,29]. However, where MCP-C focuses on the caregiver, CASA integrates meaning with Latino culture, communication, and dyadic elements (patients and caregivers simultaneously). Caregiver–Patient Support to Latinx Coping with Advanced Cancer (CASA) is an MCP-L adaptation aimed toward Latino advanced cancer patients and their caregivers as a dyad. The initial phase of this adaption included the patient and caregiver’s initial evaluation of the intervention’s protocols. CASA’s cultural adaptation integrated MCP concepts and communication skills training (CST). Patients and caregivers reported acceptance and goals of MCP and CST, feasibility, and therapeutic method. For more information regarding CASA, please access the authors’ intervention acceptability and feasibility publication [30].

Caregivers play an integral role in advanced cancer patients’ trajectory. Often, they are present from the diagnosis to their death [12]. They witness at first hand their loved one’s needs, as well as their own, highlighting the need to adapt interventions which integrate both patient and caregiver culturally. This makes their contribution all more essential when adapting a cultural adaptation of a psychosocial intervention. The main goal of this article is to present a case study of a Latino advanced former cancer caregiver experience with a focus on the acceptance of Caregiver–Patient Support to Latinx Coping with Advanced Cancer (CASA) intervention. This case study will provide this former caregiver’s acceptability through their own perspective and provide context and a greater understanding of the dyadic experience.

## 2. Materials and Methods

### 2.1. Recruitment

A case study of a participant from the pilot study of the cultural adaptation of CASA [30,31,32] is presented, focusing on the comprehension and acceptance of the sources of the meaning of the Latino advanced cancer caregiver’s experience. The Ponce Research Institute Institutional Review Board (IRB) and Ethical Committee approved all the study procedures. An IRB-approved introductory letter introduced potential participants to the study. Recruitment was performed through the “Integrated Psychosocial Support Program or PAPSI for its Spanish acronym. PAPSI is a psychosocial support program integrated into oncology care based in the southern area of Puerto Rico. Advanced cancer patients who completed PAPSI’s routine distress screening and their family caregiver(s) were invited to participate in the study.

Patient–caregivers’ inclusion criteria were to be Latino (a person born in Latin America) and obtain a score of >3 on the Distress Thermometer. The Distress Thermometer is used to screen for emotional distress and physical symptoms among cancer patients [33]. An in-person research staff member followed up with potential participants to provide information, answer questions, and determine eligibility, including the Distress Thermometer. Those eligible and interested completed informed consent. For those who gave consent, a call was scheduled to complete the questionnaire and interview. The information provided in this interview provided content and information that expands on MCP and CST concepts.

Case studies allow researchers to present unique cases and use them when there is a desire to tackle an issue in-depth. The format enables exploration, describing, or explaining an occurrence in a real-life or everyday context presentation. It allows a detailed inquiry and describes a participant’s attitude and experiences. It is a method that is more suited to capturing information descriptively as opposed to a survey or experimental design [34,35]. In the parent study [30,31], multiple caregivers were interviewed, and most participants were very concise in their interview approach. However, the research team decided to explore the present subject’s data as he was more elaborate when answering our questions, providing us with deeper insight into his experience as a caregiver.

### 2.2. Measures

Measures used include sociodemographic questionnaires, standardized assessments, and a semi-structured interview. The sociodemographic questionnaire included general demographic questions (e.g., age, education, gender, and education level). The cross-sectional survey included assessments that measured caregiver burden, depression, anxiety, hopelessness, quality of life, and distress. The semi-structured interview describes the CASA intervention content and includes five sections: (1) purpose and goals; (2) intervention content—MCP and CST; (3) homework; (4) other possible topics for discussion; and (5) intervention format.

### 2.3. Scales

#### 2.3.1. Caregiver Burden

The Zarit Burden Interview is a 5-point Likert scale that assesses burden on caregivers. Scores ranging from 0–21 indicate little or no burden, scores from 21–40 represent mild to moderate burden, scores from 41–60 indicate moderate to severe burden, and scores ranging from 61–88 indicate severe burden [36,37,38,39,40].

#### 2.3.2. Depression and Anxiety

The Hospital Anxiety and Depression Scale (HADS). Scores ranging from 0–7 indicate ‘normal’ symptomatology, 8–10 indicate ‘borderline’ symptomatology, and 11 or more represent significant psychological morbidity [41,42].

#### 2.3.3. Hopelessness

The Beck Hopelessness Scale (BHS) assesses the degree of hopelessness. Scores that range from 0–3 are within a normal range, scores from 4–8 represent mild hopelessness, scores from 9–14 represent moderate hopelessness, and a score of 14 or more represents severe hopelessness [43,44].

#### 2.3.4. Distress

NCCN Distress Thermometer and Problem List is a rapid screening tool for assessing psychological distress in people affected by cancer. The tool uses a 1–10 scale to describe distress. One being little to no distress and 10 representing the maximum amount of distress the person can experience [45,46,47,48].

### 2.4. Analysis

A qualitative deductive analysis was performed with the software ATLAS.ti. Deductive categories were developed and defined a priori following standard deductive analysis procedures [49,50] and with multiple coders (minimum of three). The caregiver’s comprehension and acceptance of the intervention’s content were evaluated using the transcription of the semi-structured interview. The codes included high, moderate, low, and ambiguous comprehension and high, moderate, low, and ambiguous acceptance. To improve validity and rigor [51], we discussed the codes until a consensus was reached. To illustrate and summarize the findings, data from the semi-structured interview were quantified [49,50] and summarized in the discussion of participants’ answers.

## 3. Results

Mr. X is a 25-year-old male former caregiver. At the time of the interview, he indicated being single, unemployed, and had a high school diploma. He assumed a caregiver role for three nuclear family members who faced a cancer diagnosis. Firstly, he cared for his aunt and mother, who succumbed to cancer. Afterward, his grandfather was diagnosed and passed away due to his diagnosis. Out of the caregivers interviewed, Mr. X was chosen due to the richness of his answers. He provided background and abundant answers, which allowed the team to obtain a broader sense of what he observed in his loved one’s experiences. Moreover, he cared for three family members at a very young age and expressed extreme maturity during the interview.

On the Hospital Anxiety and Depression Scale (HADS), caregiver results show a score of two on the depression portion and four on the anxiety portion of the assessment tool. Scores indicate that the caregiver presents non-symptomatology related to depression and normal anxiety symptomatology. Scores on the Beck Hopelessness Scale show a score of seven on the hopelessness scale, which indicates mild feelings of hopelessness. Scores on the NCCN Distress Thermometer and Problem List show an auto-reported distress score of four, considered a score that presents distress. The Zarit Burden Interview results show a score of six on the caregiver burden scale. Scores indicate that the caregiver presents little to no caregiver burden. See Table 1 for results.

### 3.1. Purpose of MCP and CASA Intervention

Interviewer NTB presented the purpose of the intervention, which is to help patients and caregivers and meaning in life after a cancer diagnosis. Additionally, she broadly presented the use of communication strategies to help the dyad share their emotions and feelings to facilitate how each other may express possible sources of meaning before and after diagnosis and any decision-making. Mr. X expressed high acceptability of the intervention. He also highlighted the importance of expert-led interventions. He underscored how every patient reacts differently to a cancer diagnosis and how some may “emotionally suffer more than others”.

### 3.2. Creative Sources of Meaning

Following the purpose of the intervention, Mr. X was presented with the themes of “the will to meaning”, “freedom of will”, and “life has a meaning”. “The will to meaning” is the need to find meaning in our own experience, and it is a fundamental motivational force that humans have. Some situations might cause someone to lose the will to meaning, but there are ways to recover it. Giving life meaning is a characteristic that defines human beings. The “Life has meaning” theme highlights how life has meaning, and this never ceases to be. In cases where meaning seems lost, the potential for meaning is always present. There is always a possibility to create or experience the meaning of life through our life. “Freedom of will” is a concept that states how, as human beings, we have the freedom to find meaning in our lives and choose how to face our suffering, even when our will is taken away. Depending on the situation, we may have little to no control, but we can still choose our attitude while facing our suffering.

During this period, Mr. X verbalized high acceptability of MCP themes and introduced his family history. He shared that his deceased close family members all died from a cancer diagnosis. He used one of the family members (male) as an example of why some may or may not have free will to choose how they feel and handle a cancer diagnosis. Before the cancer diagnosis, his grandfather used to be a righteous and strong person, the type of person that never shed a tear. However, he was always in pain and vomiting due to cancer and treatments. Mr. X narrated how he witnessed his grandfather tell his wife how he did not have the desire to live. Mr. X says: “This hit him hard, and the reason is that just as it came for his two daughters, it came for him. In the end, he stated: ‘As I understand it, any person has that right or free will… it is not easy, and not everyone can achieve it.’. He believes the feeling is too strong that some cancer patients may think, ‘I do not want to keep going, I do not want to continue with this. I cannot find the meaning in life because this pain is too immense, and I cannot stand it’. He also added the experience of another family member; his mother always found meaning in her life and still found meaning after her cancer diagnosis through religion and faith. He closed this segment by expressing the subject’s delicacy and how cancer patients may interpret it differently. This must not be stated as fact, but it may be harder for some than others”.

### 3.3. Communication Strategies: Speaker and Listener

After being presented with the main themes and concepts in MCP, the interviewer presented the main communication points between the speaker and listener. The two main objectives of a conversation are to share feelings and emotions and decision-making or problem resolution. The couple must not be distracted, so location, setting, and time must be considered to share these feelings and emotions adequately. The speaker must speak from their point of view and use “I” when they speak. Additionally, the speaker must be honest with their feelings and emotions. They can choose what information to disclose or not disclose, but they must not provide misinformation. Finally, the person should speak in paragraphs, so they can provide the listener the opportunity to respond to the main points. The listener should show the speaker they understand what is being said and accept their right to have these feelings or thoughts. The listener can show acceptance with their posture, tone of voice, and facial expression. While the other person is speaking, the listener should put themselves in the other person’s situation and try to understand their perspective. Once the speaker finishes, the listener should resume the speaker’s thoughts, emotions, wishes, and conflicts. During the process of listening, one must not judge what is being said, express an opinion, try to solve the problem and ask questions (unless it is to clarify).

Mr. X expressed high acceptability and the importance of teaching these strategies to families, so they can listen to how the patient is feeling and their mental state. He also stated how crucial these strategies are for the patient because they are expressing themselves, which takes a toll on them. As a response to speaker strategies, Mr. X recounted how his grandmother did not like to talk about cancer. However, after meeting with the interviewer, the spouse answered questions and expressed her feelings, and as a result, she cried. However, she found it was beneficial because “she let everything go”. He additionally stated how teaching these strategies to the family and patient would be highly beneficial. Regarding listener strategies, Mr. X underscored the importance of these strategies, specifically when not to interrupt and not to make the speaker feel uncomfortable. Additionally, he expressed, “It is a difficult situation. Even if we try to put ourselves in their shoes, sometimes it is impossible to do it. Which is why we must try to comprehend it as best we can and accept the information to try and help”.

### 3.4. Cancer Meaning Identity before and after Diagnosis

Mr. X was presented with additional themes that will be discussed during the intervention: identity, creative sources of meaning, and experiential sources of meaning. Identity is influenced by roles, other people, and additional aspects that give life meaning. There is a strong relationship between identity and meaning; identity is made up of things that give meaning to a person’s life. Creative sources of meaning can be experienced by actively participating in life through work, actions, and achievements by way of compromise, courage, and responsibility. Experiential sources of meaning integrate how people connect with life through love, relationships, beauty, nature, and humor.

Mr. X expressed moderate acceptability, and the reason behind this is as follows: he observed the identity and course of two of his family members. He firstly stated: “Wow. That one is tough, it is tough. When I speak to you, I explain what I think and what I know, with my experience, what I have lived, with my family members”. The grandfather owned a workshop, working eight hours a day, six days a week. Mr. X stated: “It was his life, that is what he did every day, and it is what defined him”. Once he was diagnosed with cancer, he could not work, but he still went to his workshop to observe. When cancer worsened, he could no longer visit his workshop, and his identity was affected. As indicated by Mr. X: “I do not know any other way he was able to find the meaning of life after that. Towards the end, he did not know what to do; he stayed in his room and watched the walls. That feeling that identity he had, he did not see it anymore… he lost it.” He added that the experience was harrowing and a challenging subject for him. Additionally, Mr. X presented the case of his mother and how she kept her identity through God and her religious beliefs. He reiterates how an intervention like this could have helped him see things through another perspective and possibly assist him in redefining his identity. He also expressed how important it is for caregivers to know this information, so they can help with the process of finding a new identity. He finalized by stating how it was the first time he had talked about identity and he had never thought about it before. It has helped him contextualize and conceptualize his family member’s processes.

### 3.5. Encountering Life’s Limitations

After explaining the components of the intervention, the interviewer presented homework that would be assigned to the participants. During this assignment, the patient will be asked to answer questions about their limitations in life, losses, and obstacles they have had to deal with and how they faced them. Additionally, they will be asked to answer what limitations or losses they have had after their diagnosis, how they faced them, and if they are still able to find meaning in their daily life. Finally, patients were asked to answer what they think a “good” or “meaningful” death is and how they think loved ones will remember them.

Mr. X expressed high acceptability of the assignment. He also explained how patients might perceive questions regarding death as already being “gone from this world and being lost”. It might make them think about how they should and could die. He believed it is more appropriate to discuss how to be remembered by family members.

### 3.6. Share Your Legacy and Tell Your Story

The interviewer presented another exercise that will be assigned to the participants. During this assignment, patients will be asked to share their life stories with loved ones in a way that makes them feel comfortable. The key is to emphasize experiences that have been sources of pride and have been meaningful or things they wish to do. Additionally, the caregivers will reflect on the patient’s experiences.

Mr. X expressed: “It is always good to remember the past in a good and beautiful way”. He also explained how the patient might feel pride in things they achieved when they were young and how the caregiver feels happy when listening to the stories. He stated how his grandfather felt happy and laughed when reminiscing with the rest of his family. However, he expressed how this might be a slippery slope, and some might make the comparison between what they were and where they are now. A moment of happiness and nostalgia might bring about sadness.

### 3.7. Legacy Project: “Life as a Living Legacy”

Mr. X was presented with an activity that will be assigned to the participants. During this assignment, patients, alongside caregivers, will be asked to create a project integrating ideas presented throughout the intervention. For example, the projects could be photo albums and videos, creating music playlists, performing something they have always wanted to complete, fixing estranged relationships, and more.

Mr. X indicated high acceptability of this activity. Moreover, he stated that this is a good idea and that patients can choose to “leave with a bang” and perform something they have never performed. Regarding fixing estranged relationships, he presented an anecdote where his grandfather and sibling had a conflict with another family member and had not spoken in years. The grandfather died; however, the sibling and the other family member made amends in his honor. Even though his grandfather died, he understands the power making amends can have on a person.

### 3.8. Connecting with Life

The interviewer presented the last homework that will be assigned to the participants. During this assignment, patients and caregivers will be asked to mention three ways they connect with life and feel alive through the experiential sources of love, beauty, and humor. Mr. X indicated high acceptability and how humor is essential, especially with family. He additionally stated: “Love is the most important of them all. The love of the family and love for your partner”.

## 4. Discussion

The present case study aimed to present the content of a culturally adapted psychosocial intervention through a former cancer caregiver’s experience. Scales were used to assess caregiver symptoms of anxiety, depression, distress, caregiver burden, and hopelessness. Additionally, a semi-structured interview was used to determine the caregiver’s acceptability of the intervention. Caregiver–Patient Support to Latinx Coping with Advanced Cancer (CASA) is aimed at caregivers and their loved ones coping with cancer. Said intervention utilizes the themes of the meaning of life, will find meaning, freedom of will, and sources of meaning to assist patients and their caregivers in searching for meaning while battling cancer. Results from this case study show a former caregiver’s acceptance of a culturally adapted intervention for Latino patients and their caregivers.

Results indicate how the caregiver presents little to no symptoms of depression, anxiety, caregiver burden, and hopelessness. These results contradict the literature, suggesting that former cancer caregivers experience most of the symptoms mentioned above up to a year after the loss of a loved one [52,53]. The results could be attributed to family cohesiveness within the caregiver’s family. *Familismo* is an integral part of Latino culture, describing loyalty and the importance of family [54]. Moreover, it serves as a protective factor among this demographic [55]. A study shows how a positive family relationship may serve as a moderator between anxiety and depression [56]. Additionally, another study indicates how poor family cohesion and high family conflict are associated with higher depression [57]. However, he presented levels of distress, which is congruent with the current literature, which illustrates how former or bereaved caregivers are at risk of experiencing psychological distress [58,59].

The former caregiver provided anecdotes on the content of the interventions and how he believed they might be beneficial. Though content is meant for dyads and the caregiver has assumed the role three times, his narrative focused on the patients and did not include himself. Additional findings show how the caregiver found most of the intervention content acceptable. Said content includes the intervention purpose, communication strategies between dyads, identity before and after the cancer diagnosis, limitations after diagnosis, being remembered by loved ones, legacy projects, and connecting with life. However, he expressed mixed feelings about the themes of free will, identity, remembering the past, and dignified or good death. This moderate acceptability could be attributed to the fact that he observed the themes from a patient’s perspective and how they might negatively perceive it. The literature highlights the difference in advanced cancer patients’ experience, perspectives, challenges [60], and self-management [61], underscoring the importance of different patient and caregiver perspectives when adapting a psychosocial intervention.

## 5. Conclusions

The findings within this case study present a male caregiver and his thoughts on a culturally adapted psychosocial intervention by commenting on his experience as a caregiver for more than one family member. The caregiver shared and understood what his family members with a cancer diagnosis went through while discussing the intervention’s themes. While every caregiver’s experience differs, many share a similar journey with their loved ones. His overall acceptability of a dyad-based intervention provides an in-depth personal account of complex knowledge and underlines the reasons why the CASA intervention was deemed acceptable and feasible in the parent study. Alongside the team’s published works, these results will assist in the refinement and piloting of a psychosocial intervention for advanced Latino cancer patients and their caregivers. Specifically, it will assist providers who have patients with multiple caregiver experiences. For the future pilot study, this case study supports the need and absence of care for Latino caregivers who cope with the cancer experience without available resources. Particularly, it supports the gap in psychosocial services and the need to keep the caregiver dyadic in the process of finding meaning and improving communication skills, end-of-life care, and quality of life. As this is a case study, his experience cannot be generalized; however, his experience provides insight that will contribute to CASA’s adaptation and implementation.

## Figures and Tables

**Table 1:** Caregiver psychosocial symptoms.

Assessment	Score
Hospital Anxiety and Depression Scale	
Anxiety	4
Depression	2
Beck Hopelessness Scale	7
NCCN Distress Thermometer (DT)	4
Zarit Caregiver Burden	6

## Data Availability

Not Applicable.
